# Hierarchical Evaluation of Predictive Models for Confirmed Sarcopenia: Discrimination, Calibration, and Clinical Applicability in a Cross-Sectional Study of Older Adults

**DOI:** 10.3390/jcm14248707

**Published:** 2025-12-09

**Authors:** Ludwig Álvarez-Córdova, Daniel Simancas-Racines, Claudia Reytor-González, Diana Fonseca-Pérez, Víctor Sierra-Nieto, Cecilia Arteaga-Pazmiño, Natasha Giler-Párraga, Jaen Cagua-Ordoñez, Martha Montalvan

**Affiliations:** 1 Maestría de Nutrición y Dietética, Facultad de Ciencias de la Salud, Universidad de las Américas (UDLA), Quito 170124, Ecuador; 2 Facultad de Salud y Bienestar, Pontificia Universidad Católica del Ecuador, Quito 170143, Ecuador; danielsimancas10@gmail.com; 3 Escuela de Medicina, Pontificia Universidad Católica del Ecuador, Santo Domingo 230203, Ecuador; claudiareytor@gmail.com (C.R.-G.);; 4 Carrera de Nutrición y Dietética, Facultad de Ciencias de la Salud, Universidad Católica De Santiago de Guayaquil, Guayaquil 090614, Ecuador; 5 Carrera de Fisioterapia, Facultad de Ciencias de la Salud, Universidad Católica De Santiago de Guayaquil, Guayaquil 090614, Ecuador; 6 Carrera de Nutrición y Dietética, Facultad de Ciencias Médicas, Universidad de Guayaquil, Guayaquil 090510, Ecuador; 7 School of Medicine, Universidad Espíritu Santo, Samborondón 0901952, Ecuador

**Keywords:** sarcopenia, older adults, predictive models, calf circumference, mini nutritional assessment, phase angle

## Abstract

**Background**: Sarcopenia is a progressive and multifactorial condition linked to aging, malnutrition, and chronic diseases, presenting significant clinical and public health challenges. Current screening tools vary in complexity and diagnostic accuracy, emphasizing the need for simple, evidence-based predictive models suitable for settings with limited resources. **Methods**: A cross-sectional study was conducted among community-dwelling older adults to develop and internally validate hierarchical predictive models for sarcopenia using readily available primary care variables. Three models were built: (1) a basic clinical model (age, sex, BMI, calf circumference, and SARC-F), (2) a model including nutritional status (Mini Nutritional Assessment, MNA), and (3) an extended model adding bioelectrical impedance parameters (phase angle, PhA). Model performance was assessed using AUC, Brier score, Hosmer–Lemeshow test, and decision curve analysis. **Results**: The parsimonious model demonstrated excellent discrimination (AUC = 0.91) and good calibration (Hosmer–Lemeshow *p* = 0.36), while the extended model with MNA and PhA achieved the highest overall performance (AUC = 0.95; Brier = 0.064; *p* = 0.97). Incorporating MNA and PhA enhanced calibration and clinical utility, especially for risk probabilities between 0.10 and 0.40. Internal validation showed minimal optimism and stable coefficients, with BMI, sex, and PhA as consistent predictors. **Conclusions**: A model combining anthropometric, nutritional, and bioelectrical variables provides high diagnostic accuracy for sarcopenia while remaining practical for clinical use. Its stepwise design facilitates application at various healthcare levels, supporting early detection and targeted interventions in aging populations.

## 1. Introduction

Sarcopenia, a progressive and generalized loss of skeletal muscle mass and strength, represents a core component of frailty and is now recognized as a significant public health challenge in aging populations worldwide [1]. Its prevalence rises markedly with age, reaching 10–27% among community-dwelling older adults and exceeding 40% in hospitalized or institutionalized populations [2,3]. Beyond its musculoskeletal manifestations, sarcopenia contributes to functional decline, increased risk of falls, loss of independence, and higher morbidity and mortality rates, making it one of the most impactful geriatric syndromes in modern health systems [4,5].

The conceptual understanding of sarcopenia has evolved substantially over the past decade [3]. The European Working Group on Sarcopenia in Older People (EWGSOP2) redefined the condition as a muscle disease (muscle failure) characterized by impaired strength as its primary indicator, supported by low muscle quantity or quality [6,7,8]. Despite the availability of standardized diagnostic criteria, the clinical implementation remains suboptimal, largely due to methodological heterogeneity and limited access to specialized measurements such as dual-energy X-ray absorptiometry (DXA) or magnetic resonance imaging (MRI), which are considered reference techniques for quantifying muscle mass [9].

As a consequence, there is an urgent need for feasible, valid, and scalable diagnostic alternatives that can be applied in both community and clinical settings [10], particularly in low- and middle-income countries where technological and infrastructural constraints limit comprehensive evaluation. Screening tools such as strength, assistance with walking, rising from a chair, climbing stairs and falls (SARC-F) questionnaire have been proposed to address this gap [11], but their sensitivity is often poor when used in isolation [12]. Similarly, single anthropometric markers, such as calf circumference, serve as practical proxies for muscle mass but may not capture the multidimensional nature of sarcopenia.

Recent studies have highlighted the potential of multivariable predictive models that combine functional, anthropometric, nutritional, and bioelectrical indicators to improve diagnostic precision [13,14,15]. Bioelectrical impedance analysis (BIA), for instance, allows the estimation of phase angle (PhA)—a non-invasive marker of cellular integrity and muscle quality [16,17,18]—while nutritional assessments such as the Mini Nutritional Assessment (MNA) capture metabolic and dietary factors closely linked to muscle preservation [19,20].

However, comparative evidence quantifying the incremental diagnostic contribution of these domains remains limited, and few investigations have systematically evaluated their hierarchical integration into predictive frameworks.

Furthermore, the Latin American region context is notably underrepresented in sarcopenia research, despite the region’s rapidly aging demographic and high prevalence of chronic diseases that accelerate muscle loss [21]. Understanding the relative diagnostic value of simple versus advanced clinical indicators is essential for developing context-appropriate algorithms that balance accuracy, cost, and feasibility.

Given this background, the present study aimed to evaluate and compare the diagnostic performance, calibration, and clinical applicability of hierarchical predictive models for diagnosing confirmed sarcopenia in older adults. By progressively integrating functional (SARC-F), anthropometric (body mass index (BMI), calf circumference (CC)), nutritional (MNA), and bioelectrical (PhA) domains, this research sought to quantify the incremental gain in predictive accuracy across model configurations. The ultimate goal was to identify parsimonious models capable of supporting early, reliable, and context-sensitive detection of sarcopenia—bridging the gap between population screening and specialized clinical diagnosis.

## 2. Methods

### 2.1. Study Design and Setting

A cross-sectional analytical study was conducted to evaluate the diagnostic accuracy, calibration, and clinical utility of hierarchical predictive models for confirmed sarcopenia in older adults. Data were extracted from a prospectively maintained clinical database that included anthropometric, functional, nutritional, and bioelectrical measurements obtained in hospital and outpatient settings in Ecuador. The study adhered to standardized reporting frameworks, including TRIPOD for prediction models [22], PROBAST for assessing risk of bias and applicability [23], and the STROBE statement for observational studies [24].

### 2.2. Population and Eligibility Criteria

Participants were adults aged ≥50 years with complete data on handgrip strength, body composition assessed by bioelectrical impedance analysis (BIA), and SARC-F screening. Inclusion criteria required: (1) age ≥ 50 years; (2) availability of valid measurements for muscle strength, muscle mass (Appendicular Skeletal Muscle Mass [ASM] or Fat-Free Mass Index [FFMI]), and SARC-F; and (3) stable clinical condition. Exclusion criteria included: (1) limb amputation, edema, ascites, or metallic implants interfering with BIA; (2) active malignancy, terminal illness, or stage IV–V chronic kidney disease; and (3) incomplete or implausible anthropometric or BIA data. Only participants with complete information for all variables included in the hierarchical models were analyzed to ensure comparability across model configurations.

### 2.3. Data Sources and Measurement Procedures

All measurements adhered to internationally accepted standards to ensure accuracy and reproducibility. Assessments were performed in a temperature-controlled environment (22–25 °C) during morning hours, after at least four hours of fasting and bladder emptying to reduce fluid-related variability in BIA results.

Anthropometric measurements followed the protocols set by the International Society for the Advancement of Kinanthropometry (ISAK). Body weight was measured using a calibrated digital scale (accuracy 0.1 kg), and height was recorded barefoot with a wall-mounted stadiometer (accuracy 0.1 cm). BMI was calculated as weight (kg) divided by height squared (m^2^). CC was measured at the maximum circumference of the gastrocnemius muscle, and waist and hip circumferences were taken at the narrowest and widest points of the abdomen, respectively, to determine the waist-to-hip ratio (WHR).

Muscle strength was measured using handgrip dynamometry following EWGSOP2 guidelines. Participants sat with their elbows bent at 90° and their wrists in a neutral position. Three attempts were made on the dominant hand, with 60 s rest between trials; the highest value in kilograms was recorded. Low muscle strength was defined as <27 kg for men and <16 kg for women.

Body composition was measured using multifrequency BIA (50 kHz reference, tetrapolar setup), following the guidelines of the European Society for Clinical Nutrition and Metabolism (ESPEN). Resistance (R) and reactance (Xc) were recorded to calculate the phase angle (PhA, in degrees):

PhA~(^∘=arctan{├(⍁{X_c}{R}┤)}×├(⍁{180}{π}┤)

Derived indices included fat-free mass (FFM), fat mass (FM), ASM, FFMI, and fat mass index (FMI), all standardized by height^2^ (m^2^). Functional performance was evaluated using the Short Physical Performance Battery (SPPB), comprising balance, gait speed (4 m), and chair rise tests. Scores range from 0 to 12, with <8 indicating low performance.

Nutritional status was assessed via the MNA, which integrates dietary intake, weight loss, mobility, psychological stress, and BMI. Scores were classified as: <17 (malnutrition), 17–23.5 (risk of malnutrition), and ≥24 (normal). Sarcopenia screening employed the SARC-F questionnaire, consisting of five self-reported items (strength, walking assistance, chair rise, stair climbing, and falls), each scored 0–2, with a total score ≥4 indicating risk.

All data underwent internal quality control, including double data entry and cross-verification. Measurement error across operators was maintained below 2% for anthropometry and below 1 Ω for impedance readings.

### 2.4. Variables and Operational Definitions

Confirmed sarcopenia—the primary outcome—was defined by EWGSOP2 criteria, integrating low muscle strength and low muscle mass. Muscle mass (ASM, kg) was estimated via the Sergi et al. (2015) equation [25]:

$$ASM\sim \left(kg\right)=0.401\times \left(\frac{{\left[\mathrm{Height}\;\left(\mathrm{cm}\right)\right]}^{2}}{\mathrm{Resistance}\;t\;50\;\mathrm{Hz}}\right)+\left(3.825\times \mathrm{Sex}\right)-\left(0.071\times \mathrm{Age}\right)+5.102$$

where sex = 1 for men and 0 for women. ASM was standardized by height squared to calculate the ASM index (kg/m^2^). Low muscle mass was defined using EWGSOP2 thresholds of <7.0 kg/m^2^ for men and <5.5 kg/m^2^ for women. Confirmed sarcopenia was defined as the coexistence of low muscle strength and low muscle mass (coded as 1 = present, 0 = absent).

For models, all continuous predictors (age, BMI, CC, MNA, PhA) were kept in their original scales. Categorical predictors (sex, comorbidities) were coded as binary variables. Derived indices (FMI, FFMI, %FM) were used descriptively but not included in final models to prevent collinearity.

### 2.5. Statistical Analysis

#### 2.5.1. Descriptive and Bivariate Analysis

Continuous variables were summarized as mean ± standard deviation (SD) or median (interquartile range (IQR)), based on their distribution, which was evaluated through the Shapiro–Wilk test and inspection of Q–Q plots. Categorical variables were expressed as absolute frequencies and percentages. Between-group comparisons (with vs. without confirmed sarcopenia) were performed using the *t*-test or Mann–Whitney U test for continuous variables and the χ^2^ or Fisher’s exact test for categorical variables. Candidate predictors considered for multivariable modeling were based on biological plausibility and a liberal significance threshold (*p* < 0.20) observed in bivariate analyses. Multicollinearity was assessed using the Variance Inflation Factor (VIF), with values > 5 indicating excessive redundancy.

#### 2.5.2. Hierarchical Model Construction

The explicit null hypothesis was that adding nutritional (MNA) and bioelectrical (Phase Angle) variables to the basic clinical model (age, sex, BMI, calf circumference, SARC-F) would not produce significant improvements in discrimination, calibration, or clinical utility.

To assess the additional diagnostic value of clinical, anthropometric, nutritional, and bioelectrical factors, five hierarchical logistic regression models were built sequentially. Each model calculated the log-odds of confirmed sarcopenia (*p*) using the logistic equation:

$$log\left(\frac{p}{1-p}\right)=\beta_{0}+\beta_{1}X_{1}+\beta_{2}X_{2}+\cdots +\beta_{k}X_{k}$$

The models were defined as follows:

Model 1—SARC-F (Screening model): SARC-F total score only.

Model 2—SARC-Calf (Dual model): SARC-F plus CC.

Model 3—Parsimonious Clinical Model: age, sex, BMI, CC, and SARC-F.

Model 4—Extended Nutritional–Cellular Model: Model 3 plus MNA and PhA.

Model 5—Adjusted Model: Model 3 plus comorbidities (hypertension, diabetes, and dyslipidemia).

### 2.6. Model Diagnostics and Assumption Testing

All models were checked for logistic regression assumptions. The linearity of the logit for continuous variables (age, BMI, CC, MNA, and PhA) was verified using the Box–Tidwell transformation, and nonlinearity was addressed by applying restricted cubic splines with three knots. Multicollinearity was assessed with VIFs and correlation matrices to ensure predictors were independent. Influential observations were identified using Cook’s distance (D_i_ > 4/n) and leverage (h_i_ > 2 (k + 1)/n); their effects were evaluated through sensitivity analyses.

Model calibration was planned to be evaluated using the Hosmer–Lemeshow goodness-of-fit test (*p* > 0.05 indicating adequate fit) and calibration plots comparing observed versus predicted probabilities. Model discrimination was quantified by the area under the receiver operating characteristic curve (AUC), interpreted as poor (0.5–0.6), acceptable (0.6–0.75), good (0.75–0.9), or excellent (>0.9). Pairwise DeLong’s tests for correlated receiver operating characteristic (ROC) curves were applied to compare discrimination between successive models.

### 2.7. Validation and Performance Metrics

Internal validation was conducted through bootstrap resampling with 1000 iterations to estimate optimism-corrected AUCs and assess model stability. Predictive accuracy was measured using the Brier score, which is the mean squared difference between predicted probabilities and observed outcomes (lower scores indicate better accuracy). Calibration intercepts and slopes were also calculated in bootstrap-adjusted models to examine systematic under- or overestimation of predicted risk. Threshold-based performance metrics were determined using Youden’s index to find the optimal probability cut-off that maximizes sensitivity and specificity. Diagnostic measures—including sensitivity, specificity, positive predictive value (PPV), and negative predictive value (NPV)—were calculated for each model at its optimal threshold.

### 2.8. Sensitivity, Subgroup, and Robustness Analyses

Sensitivity analyses were performed excluding participants with extreme BMI values (<18.5 or >35 kg/m^2^) and those with incomplete secondary variables. Subgroup analyses stratified by sex, age group (<75 vs. ≥75 years), and nutritional status (MNA < 24 vs. ≥24) were used to evaluate model consistency across groups. Nonlinear relationships for continuous variables were further examined with restricted cubic splines. Residual and influence diagnostics were conducted to ensure no heteroscedasticity or outliers that could impact model stability.

### 2.9. Model Selection and Clinical Interpretation

The comparative performance of hierarchical models was summarized using ΔAUC, ΔBrier, and DeLong *p*-values to assess incremental diagnostic improvements. Clinical feasibility was also evaluated, focusing on the practicality of collecting variables and their applicability in various healthcare settings. Models that require only anthropometric and screening variables were deemed suitable for community or primary care, while those incorporating nutritional and bioelectrical parameters were considered more appropriate for specialized clinical assessments.

### 2.10. Statistical Threshold and Software

All analyses were conducted using R version 4.5.0 (R Foundation for Statistical Computing, Vienna, Austria). Statistical significance was defined as a two-tailed *p* < 0.05, and all estimates are presented with 95% confidence intervals (CI). The analytical approach aimed to compare the discrimination, calibration, and overall predictive accuracy of five hierarchical logistic regression models developed to identify confirmed sarcopenia.

### 2.11. Ethical Considerations

The database was anonymized prior to analysis to maintain confidentiality. The study followed the ethical principles of the Declaration of Helsinki (2013) and was reviewed and approved by the Institutional Research Ethics Committee of the participating institution.

## 3. Results

### 3.1. Descriptive Analysis

A total of 246 adults aged 50 years or older were included in the analysis, of whom 35 (14.2%) met the EWGSOP2 criteria for confirmed sarcopenia. Participants with sarcopenia were significantly older (median 80.0 [71.0–88.0] years) than those without sarcopenia (72.0 [65.0–80.0] years, *p* < 0.001). BMI was notably lower among individuals with sarcopenia (22.3 ± 2.8 kg/m^2^) compared to those without (28.8 ± 4.8 kg/m^2^, *p* < 0.001). Similarly, CC was smaller (30.1 [27.6–32.4] cm vs. 35.1 [31.7–37.5] cm, *p* < 0.001), and PhA values were reduced (4.4 [4.0–4.8]° vs. 5.3 [4.7–6.1]°, *p* < 0.001), consistent with decreased cellular integrity.

No significant differences were detected in the SARC-F or MNA scores (*p* = 0.491 and *p* = 0.462, respectively). Sex distribution and comorbidity frequencies were comparable (all *p* > 0.05). Overall, older age, lower BMI, reduced CC, and lower PhA distinguished participants with confirmed sarcopenia (Table 1).

### 3.2. Model Discrimination and Hierarchical Comparison

**Figure 1:**
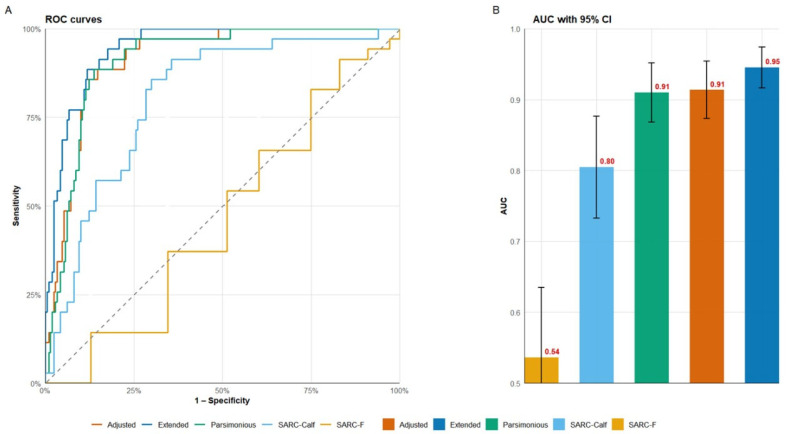
Discriminative performance and comparative accuracy of hierarchical models for confirmed sarcopenia. Panel (**A**) shows the ROC curves for the five hierarchical logistic regression models evaluating confirmed sarcopenia: SARC-F (M1), SARC-Calf (M2), Parsimonious Clinical (M3), Extended Nutritional–Cellular (M4) and Adjusted (M5). Panel (**B**) displays the AUC values with 95% confidence intervals for each model.

Progressive model construction showed a clear, consistent improvement in discrimination as physiologically informative predictors were added (Figure 1). The univariate SARC-F model (M1) had poor discrimination (AUC = 0.54; 95% CI 0.44–0.63), confirming the limited diagnostic accuracy of self-reported function alone. Adding CC, model (M2), increased the AUC by +0.27 to 0.80 (95% CI 0.73–0.88), representing a significant improvement in discrimination and highlighting the role of lower-limb muscle bulk in case separation.

The parsimonious clinical model (M3)—including age, sex, BMI, CC, and SARC-F—achieved excellent performance (AUC = 0.91; 95% CI 0.87–0.95). DeLong’s pairwise test confirmed a significant improvement over M2 (Z = −3.21, *p* = 0.001), demonstrating the synergistic diagnostic value of combining demographic and morphofunctional data.

Incorporating nutritional (MNA) and cellular (PhA) variables into the extended model (M4) further enhanced discrimination, reaching an AUC of 0.95 (95% CI 0.92–0.97), with a statistically significant improvement over M3 (ΔAUC = 0.04; Z = −2.53; *p* = 0.011). The narrow 95% CI and low variance inflation (all VIF < 2) indicate model stability and strong internal consistency.

Conversely, adjusting for cardiometabolic comorbidities (hypertension, diabetes, dyslipidemia) in the adjusted model (M5) did not improve discrimination (AUC = 0.91; 95% CI 0.87–0.95; *p* = 0.56 vs. M3). This plateau indicates that comorbidity information does not add predictive value beyond the anthropometric, nutritional, and bioelectrical factors already included in previous models.

Overall, the discrimination trajectory shows steep initial improvement from M1 to M3, a modest yet statistically significant refinement in M4, and stabilization afterward. The cumulative gain across models indicates that most diagnostic information for confirmed sarcopenia can be obtained using readily available clinical and anthropometric variables, while PhA and MNA offer an additional margin of precision.

### 3.3. Calibration and Predictive Accuracy

Calibration analysis confirmed that predicted probabilities closely matched observed event rates across all multivariable models. The Hosmer–Lemeshow goodness-of-fit test showed non-significant results for M3–M5, indicating good fit: M3 χ^2^ = 8.83 (*p* = 0.36), M4 χ^2^ = 2.40 (*p* = 0.97), M5 χ^2^ = 7.16 (*p* = 0.52).

Among these, M4 exhibited near-perfect calibration, as visual inspection of calibration plots showed minimal deviation from the 45° line, consistent with ideal probability alignment. Predictive accuracy, measured by the Brier score, improved with increasing model complexity: M3 = 0.084, M4 = 0.064, M5 = 0.080. The lower score of M4 indicates greater reliability of predicted probabilities and a reduced mean squared error between observed and predicted outcomes.

Bootstrap resampling with 1000 iterations yielded optimism-corrected AUCs that closely matched the apparent AUCs—0.896 (M3), 0.930 (M4), and 0.889 (M5)—indicating minimal overfitting and strong internal validity. The small optimism (<0.02 absolute difference) supports the reproducibility of M3–M4 in independent samples with similar demographics.

Collectively, these results confirm that both M3 and M4 achieve strong calibration and accuracy. While M4 represents the best-fitting and most stable configuration, the parsimony of M3 offers nearly equivalent performance at a reduced data-acquisition burden.

### 3.4. Model Diagnostics and Robustness

Model assumptions were checked before analysis. The linearity of the logit was confirmed for all continuous predictors (age, BMI, CC, MNA, PhA) using Box–Tidwell transformation (all *p* > 0.05), and restricted cubic spline adjustments verified no nonlinear distortions. No predictor exceeded the multicollinearity threshold (all VIF < 2.0), confirming independence among variables.

Influence diagnostics identified no cases with Cook’s distance > 4/n or leverage > 2 (k + 1)/n, indicating model stability and absence of high-leverage outliers. Residual analyses confirmed normality of the deviance residuals and homoscedasticity across the predicted probability range.

Sensitivity analyses excluding individuals with extreme BMI (<18.5 or >35 kg/m^2^) or incomplete nutritional/bioelectrical data produced nearly identical AUC values (ΔAUC < 0.01 for all models), demonstrating robustness of effect estimates.

Subgroup analyses showed no significant interaction between sex or age group and the predictive accuracy (interaction *p* > 0.10), confirming that the model’s discrimination remained consistent across demographic groups. Validation by nutritional status (MNA < 24 vs. ≥24) kept an AUC above 0.90 in both groups, supporting its generalizability.

### 3.5. Comparative Performance Synthesis

When discrimination, calibration, and prediction error were evaluated together, a clear hierarchical pattern emerged. Model 1 (SARC-F) exhibited poor discrimination (AUC = 0.54), uncalibrated predictions, and a high Brier score (>0.15). Model 2 (SARC-Calf) demonstrated a substantial improvement, with acceptable discrimination (AUC = 0.80), adequate calibration (Hosmer–Lemeshow *p* = 0.41), and reduced prediction error (Brier = 0.10). Model 3 (parsimonious) achieved excellent discrimination (AUC = 0.91), good calibration (*p* = 0.36), and accurate probability estimates (Brier = 0.084).

Model 4 (extended) represented the optimal configuration, reaching the highest discrimination (AUC = 0.95), near-perfect calibration (*p* = 0.97), and minimal prediction error (Brier = 0.064). It also showed minimal optimism and the most considerable net reclassification improvement. Model 5 (adjusted) provided no additional enhancement (AUC = 0.91, *p* = 0.52, Brier = 0.080).

Decision-curve analysis showed that Model 4 provided the greatest net clinical benefit within the probability range of 0.10 to 0.40. Meanwhile, Model 3 retained over 90% of that benefit with significantly fewer input variables. Overall, the results indicated a trend of diminishing returns: a notable diagnostic improvement between functional and anthropometric stages (M1 to M2), further enhancement with demographic and compositional covariates (M2 to M3), a modest gain with nutritional and cellular parameters (M3 to M4), and a plateau afterward (M5). From a modeling perspective, Model 3 achieved optimal parsimony, measured as the discriminative gain per added parameter. In contrast, Model 4 achieved the highest overall accuracy but required more measurement complexity, supporting a context-dependent approach for clinical use.

### 3.6. Independent Predictors Across Models

Logistic regression coefficients highlighted consistent associations across hierarchical stages. In M3, male sex (Odds ratio (OR) = 2.72; 95% CI 1.06–7.23; *p* = 0.039) and BMI (OR = 0.67; 95% CI 0.56–0.77; *p* < 0.001) were independent predictors of confirmed sarcopenia, with age, CC, and SARC-F showing non-significant trends in the expected directions.

For this model, the estimated log-odds of confirmed sarcopenia were calculated using the following equation: logit (*p*) = 7.95021 + 0.03445 (Age) + 1.00043 (Sex) − 0.40448 (BMI) − 0.07805 (Calf) − 0.07429 (SARC-F); where Sex = 1 for men and 0 for women.

The optimal probability threshold for Model 3 was 0.23 (Youden’s index). At this cut-off, the model achieved a sensitivity of 0.886, specificity of 0.863, positive predictive value of 0.52, and negative predictive value of 0.98. This performance confirms the model’s high ability to correctly rule out sarcopenia while maintaining a balanced classification strategy suitable for primary care screening.

In M4, sex remained strongly predictive (OR = 6.29; 95% CI 2.01–22.73; *p* = 0.0026), BMI maintained an inverse relationship (OR = 0.56; 95% CI 0.42–0.70; *p* < 0.001), and PhA proved to be a powerful independent factor (OR = 0.19; 95% CI 0.08–0.40; *p* < 0.001). In M5, sex (OR = 2.81; 95% CI 1.07–7.67; *p* = 0.039) and BMI (OR = 0.65; 95% CI 0.54–0.76; *p* < 0.001) remained significant, while comorbidities did not reach significance (all *p* > 0.10).

**Figure 2:**
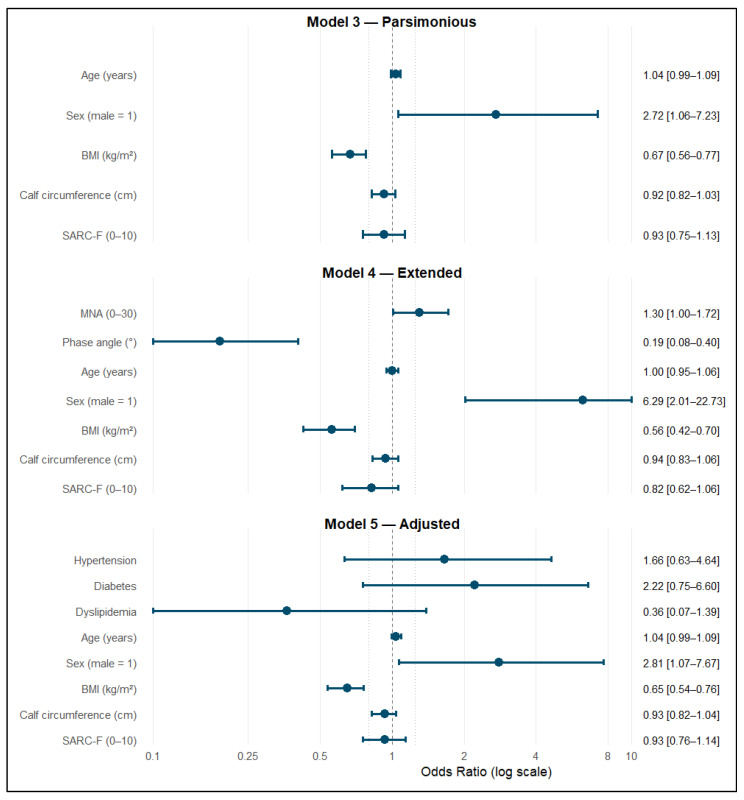
Adjusted odds ratios with 95% CI for hierarchical models predicting confirmed sarcopenia. The forest plot compares Models 3–5, displaying ORs on a logarithmic scale (0.1–10) with 95% CI. The vertical dashed line indicates the null value (OR = 1). Arrows denote truncated limits for outlying confidence intervals.

These consistent coefficients across resampling iterations reinforce parameter stability and the lack of over-parameterization. The dominance of BMI and sex across all models highlights their statistical and clinical importance within the predictive framework (Figure 2).

## 4. Discussion

Our findings show a steady and consistent enhancement in diagnostic performance as more clinically relevant domains were added. This layered pattern suggests that increased model complexity does not reduce simplicity when the added variables are truly informative. The simple model—consisting of age, sex, BMI, CC, and SARC-F—achieved excellent discrimination (AUC = 0.91), good calibration (Hosmer–Lemeshow *p* = 0.36), and low prediction error (Brier = 0.084). The expanded model—including MNA and PhA—further improved overall performance (AUC = 0.95; Brier = 0.064; H–L *p* = 0.97), with the highest clinical benefit seen for probability thresholds between 0.10 and 0.40. The gains leveled off after accounting for comorbidities, which did not add any discriminative power. Internal validation revealed minimal optimism and high coefficient stability, emphasizing BMI and sex as strong predictors and PhA as an independent factor in the extended models.

Taken together, these results reject the null hypothesis by demonstrating that the addition of nutritional (MNA) and bioelectrical (PhA) variables provides measurable improvements in discrimination and calibration beyond the basic clinical model based on age, sex, BMI, calf circumference, and SARC-F.

Interestingly, male sex emerged as a significant predictor of confirmed sarcopenia, a pattern that differs from large epidemiological studies from the United Kingdom and Korea reporting a higher burden among women [26,27]. This discrepancy likely reflects differences in study design, operational definitions (EWGSOP2 versus strength-only or mass-only criteria), and regional demographic and metabolic patterns. Further population-based research in Latin America is needed to clarify whether this male-specific association represents a true regional trend.

CC emerged as one of the most influential variables in our models, substantially increasing discrimination when added to SARC-F and the minimal clinical set. This finding aligns with recent evidence. Vanitcharoenkul et al., 2024 [28] analyzed 2455 participants from the Thai National Musculoskeletal Disease Study and concluded that SARC-F and SARC-CalF alone are inadequate for diagnosing sarcopenia. In contrast, isolated CC measurement was the best screening tool. Similarly, Álvarez-Bustos et al., 2024 [29], in 1531 community-dwelling older adults from the Toledo Study for Healthy Aging, reported an AUC of ≈0.82 for CC (EWGSOP2 cut-offs), underscoring its diagnostic and prognostic value. In a Japanese geriatric cohort, Sato et al., 2025 [30] confirmed its practical applicability, while in hospitalized frail patients, Canonico et al., 2024 [31] demonstrated that lower CC was associated with reduced in-hospital mortality (OR = 0.441; 95% CI: 0.257–0.754; *p* = 0.003). Although their study used a cohort design and ours was cross-sectional, both highlight CC as a clinically powerful marker with potential prognostic value beyond its diagnostic utility.

Evidence on SARC-F and its variants consistently shows high specificity but limited sensitivity when used in isolation. Simşek et al., 2024 [32] reported poor screening accuracy (AUC = 0.50), with minimal improvement after adding age or BMI, whereas SARC-CalF versions—particularly those using population-based cut-offs of 32/33 cm—performed better in sensitivity and AUC. A recent network meta-analysis comparing five screening tools confirmed that SARC-F exhibits very high specificity (≈99.8%) but low sensitivity, reinforcing the need to combine it with objective measures [33]. In the same line, Valencia-Muntalà et al., 2024 [34] reported variability in SARC-F performance depending on diagnostic criteria (EWGSOP1 vs. EWGSOP2) and population (older women with rheumatoid arthritis), illustrating how clinical context and gold standard influence diagnostic validity.

PhA in our models acted as an independent predictor, improving calibration and reducing prediction error when added to the parsimonious set. Two recent meta-analyses reported moderate diagnostic accuracy and proposed reference thresholds between 4.2° and 5.2° for sarcopenia detection [35,36]. Although our study did not establish cut-offs, the results agree that PhA reflects cellular and muscular quality, providing a physiological signal distinct from conventional anthropometric parameters. Koçak et al., 2024 [37] also found strong associations between PhA and components of sarcopenia within a comprehensive geriatric assessment, supporting its integration into multifactorial predictive models.

Nutritional status (MNA) also contributed to improved discrimination and calibration when incorporated into the extended model. In hospitalized populations, Yao et al., 2025 [38] showed that MNA correlated significantly with sarcopenia defined by computed tomography, while the systematic review and meta-analysis by Prokopidis et al., 2025 [20] confirmed strong associations and revealed that the coexistence of malnutrition and sarcopenia increases mortality risk over fourfold compared to sarcopenia alone. Additionally, Kammar-García, 2025 [19] compared short (SF) and long (LF) MNA forms for sarcopenia detection, reporting AUCs of 0.68 (95% CI: 0.58–0.78) and 0.60 (95% CI: 0.49–0.71), respectively, supporting MNA-SF as a feasible, efficient screening option in geriatric settings. In our study, MNA improved extended-model calibration, reflecting its potential as a comprehensive marker of metabolic and functional risk.

From a clinical standpoint, improved calibration indicates that predicted probabilities reflect the actual likelihood of confirmed sarcopenia across risk strata. This is particularly relevant for screening models, where overestimation may lead to unnecessary referrals, whereas underestimation may delay evaluation and intervention. The extended model’s near-ideal calibration slope and intercept therefore increase its utility for real-world decision-making.

Regarding predictive models, our results emphasize the balance between diagnostic precision and clinical feasibility. Li et al., 2024 [39] developed a nomogram using simple variables (age, BMI, grip strength, gait speed, and CC), yielding AUCs of 0.77 (training) and 0.76 (validation) with good calibration—results comparable to our parsimonious model, which achieved higher discrimination using equally accessible variables. Likewise, Yin et al., 2025 [15] conducted a systematic review and meta-analysis of 29 studies (70 models), finding a mean AUC of 0.91 but widespread lack of external validation and heterogeneous diagnostic criteria. They advocate prioritizing model feasibility and reproducibility over algorithmic complexity, supporting our hierarchical framework adaptable to different levels of care.

Similarly, Guo et al., 2024 [40] demonstrated that enriched SARC-F versions (SARC-F plus BMI, SARC-CalF) reached AUCs of 0.79–0.88 and sensitivities near 80%, outperforming the traditional SARC-F (AUC = 0.63). This pattern mirrors our incremental approach, where each added domain (CC, BMI, MNA, PhA) enhances discrimination without compromising operational simplicity. Meanwhile, Yu et al., 2025 [41] applied explainable machine learning (Extreme Gradient Boosting plus SHapley Additive exPlanations) and identified BMI and age as dominant predictors—consistent with our model’s most stable variables.

Collectively, comparative evidence underscores that diagnostic performance depends less on the quantity of variables and more on the rational selection of clinically relevant predictors available in routine practice. Our hierarchical model demonstrates that progressively integrating nutritional and bioelectrical domains improves calibration and reduces prediction error while maintaining clinical practicality—offering a realistic tool for early sarcopenia detection across healthcare levels.

Among the strengths, this study (i) establishes a structured, hierarchical framework quantifying the incremental benefit of each domain; (ii) applies robust internal validation with bootstrap resampling; (iii) relies on accessible clinical variables suited to primary care; and (iv) confirms model stability through sensitivity analyses by sex, age, and nutritional status.

However, several limitations must be acknowledged. The cross-sectional design precludes causal inference or longitudinal validation (e.g., hospitalization, disability, mortality). The single-center sample may limit generalizability to broader populations. The absence of imaging-based reference methods (e.g., DXA or MRI) limits direct comparison with gold standards, despite rigorous application of EWGSOP2 criteria.

In addition, the limited number of confirmed sarcopenia cases resulted in a low events-per-variable (EPV) ratio—particularly for the extended model—falling below the commonly recommended threshold of 10. According to PROBAST and TRIPOD guidance, EPV values in this range increase the risk of overfitting and may reduce coefficient stability, potentially inflating overall model performance despite the strong AUC and calibration metrics observed. In this context, the results should be interpreted with caution, acknowledging that the small number of events restricts the statistical efficiency of the regression models.

Although internal validation using 1000 bootstrap resamples helped mitigate optimism and supported the consistency of coefficient estimates, bootstrap procedures cannot fully substitute a true external validation process. Therefore, future research should test these models in independent, multicenter cohorts, ideally with higher event counts, and consider incorporating penalized regression techniques (e.g., ridge, LASSO, or global shrinkage) to enhance model robustness and generalizability.

Additionally, resampling and penalization methods commonly used to address class imbalance in predictive modeling—such as SMOTE, ROSE, or Firth’s penalized likelihood correction—were considered but intentionally not applied. Synthetic oversampling techniques can alter the natural prevalence of the outcome and compromise the interpretability of predicted probabilities in clinically oriented logistic regression models. Likewise, Firth correction is typically reserved for situations with separation or unstable coefficients, which were not observed in our analyses. Given our objective of preserving prevalence-consistent estimates and maintaining clinical interpretability, we prioritized conventional regression modeling. Nonetheless, future studies with larger and more heterogeneous samples may explore these approaches to determine whether they provide added methodological value.

Regarding the multiple pairwise comparisons performed using DeLong tests, we acknowledge the potential risk of inflated Type I error. However, formal multiplicity corrections (e.g., Bonferroni–Holm) were not applied because the hierarchical models compared in this study are not statistically independent; each model is nested within the previous one and shares overlapping predictors. In such cases, strict multiplicity adjustment may be overly conservative and could obscure clinically meaningful differences. Nonetheless, the possibility of residual Type I error cannot be fully excluded and should be considered when interpreting marginal differences in AUC values.

Furthermore, the heterogeneity of diagnostic definitions across studies, including EWGSOP2, Asian Working Group for Sarcopenia, Second Edition, and Foundation for the National Institutes of Health criteria, along with variable CC cut-offs, complicates between-study comparability. Some comorbidities were self-reported, potentially introducing residual information bias, although this did not significantly alter model discrimination.

From a clinical perspective, our findings support a stepwise implementation: the parsimonious model is suitable for primary care screening—accurate, inexpensive, and easy to apply—while the extended model incorporating MNA and PhA is better suited for specialized settings requiring refined diagnostic decisions (probability thresholds 0.10–0.40).

Additionally, implementation in real-world primary care may be facilitated by the model’s reliance on simple, low-cost measurements (age, BMI, CC, SARC-F), which can be completed within minutes and without specialized equipment. This may support early identification of at-risk patients in resource-limited environments, although workflow integration, staff training, and population-specific recalibration would be necessary for successful adoption.

From a public health standpoint, developing an adaptable algorithm tailored to Latin American contexts—where resources are constrained and populations are heterogeneous—offers a strategic opportunity to expand screening coverage, target nutritional and exercise interventions, and mitigate the disability and hospitalization burdens associated with sarcopenia. Although the hierarchical models showed strong internal performance, their external validity in populations with different demographic, nutritional, and functional profiles remains uncertain. Factors such as ethnicity, body composition patterns, dietary habits, and regional prevalence of chronic conditions may influence both coefficient stability and calibration. Therefore, model recalibration and validation across diverse Latin American and non-Latin American cohorts will be essential before broader clinical deployment.

Finally, future research should pursue multicenter external validations across diverse ethnic and socioeconomic settings, as well as longitudinal cohorts to assess prognostic capacity for adverse outcomes. Comparative studies with reference techniques (DXA, MRI) and implementation research—addressing cost-effectiveness, acceptability, and operational thresholds—will be essential for translation into real-world clinical workflows.

## 5. Conclusions

This hierarchical evaluation showed that progressively refining the model significantly enhanced its ability to predict confirmed sarcopenia in older adults. Transitioning from a symptom-based approach (SARC-F) to a clinically integrated framework that combines demographic, anthropometric, and functional measures resulted in a clear, step-by-step improvement in discrimination and calibration. The streamlined model—which includes age, sex, BMI, CC, and SARC-F—performed remarkably well (AUC = 0.91; Brier = 0.084; Hosmer–Lemeshow *p* = 0.36), while the more comprehensive model that added nutritional status (MNA) and PhA achieved even better predictive accuracy (AUC = 0.95; Brier = 0.064; Hosmer–Lemeshow *p* = 0.97).

Beyond statistical accuracy, the results show that most of the diagnostic performance can be achieved using readily available clinical variables, supporting the use of a simple model in large-scale or resource-limited screening settings. The additional benefit of nutritional and bioelectrical parameters further improves classification accuracy in specialized or research environments.

In summary, hierarchical modeling provides a robust and reproducible framework for assessing sarcopenia risk. The validated models, particularly the parsimonious configuration, offer a feasible, high-performance alternative for routine clinical implementation, bridging the gap between epidemiological assessment and practical diagnosis in aging populations.

## Figures and Tables

**Table 1:** Baseline characteristics of the population.

Characteristic	No (N = 211)	Yes (N = 35)	*p*-Value ^1^
Age (years)	72.00 [65.00, 80.00]	80.00 [71.00, 88.00]	<0.001
BMI (kg/m^2^)	28.81 ± 4.75	22.29 ± 2.75	<0.001
CC (cm)	35.10 [31.70, 37.50]	30.10 [27.60, 32.40]	<0.001
SARC-F score (0–10)	2.00 [1.00, 5.00]	2.00 [1.00, 4.00]	0.491
MNA (0–30)	11.00 [9.00, 12.00]	11.00 [9.00, 12.00]	0.462
PhA (°)	5.30 [4.70, 6.10]	4.40 [4.00, 4.80]	<0.001
Sex			0.056
Female	152 (72.0%)	19 (54.3%)	
Male	59 (28.0%)	16 (45.7%)	
Hypertension	144 (68.2%)	24 (68.6%)	>0.999
Diabetes mellitus	54 (25.6%)	10 (28.6%)	0.870
Dyslipidemia	37 (17.5%)	4 (11.4%)	0.514

^1^ Wilcoxon rank-sum test; Welch two-sample *t*-test; Pearson’s chi-squared test. Values are presented as median [Q1, Q3], mean ± SD, or n (%).

## Data Availability

The datasets analyzed during the current study are available from the corresponding author upon reasonable request. Due to participant confidentiality and institutional data protection policies, individual-level data cannot be shared publicly. Aggregated and de-identified data supporting the findings of this study will be provided upon justified request for academic or research purposes.

## Abbreviations

AUC	area under the receiver operating characteristic curve
ASM	Appendicular skeletal muscle mass
BIA	Bioelectrical impedance analysis
BMI	body mass index
CC	calf circumference
CI	confidence intervals
DXA	dual-energy X-ray absorptiometry
ESPEN	The European Society for Clinical Nutrition and Metabolism
EWGSOP2	European Working Group on Sarcopenia in Older People
FFM	fat-free mass
FFMI	fat-free mass index
FM	fat mass
IQR	interquartile range
ISAK	International Society for the Advancement of Kinanthropometry
MNA	Mini Nutritional Assessment
MRI	magnetic resonance imaging
NPV	negative predictive value
OR	Odds ratio
PhA	phase angle
PPV	positive predictive value
R	Resistance
ROC	receiver operating characteristic
SARC-F	as strength, assistance with walking, rising from a chair, climbing stairs and falls
SD	standard deviation
SPPB	Short Physical Performance Battery
STROBE	Strengthening the Reporting of Observational Studies in Epidemiology
TRIPOD	Transparent Reporting of a Multivariable Prediction Model for Individual Prognosis or Diagnosis
VIF	Variance Inflation Factor
WHR	waist-to-hip ratio
Xc	Reactance
